# Interspecific competition in germination of bird-dispersed seeds in a habitat with sparse tree vegetation in South Africa

**DOI:** 10.1186/s40529-021-00317-6

**Published:** 2021-06-10

**Authors:** L. R. Vukeya, T. M. Mokotjomela, N. J. Malebo, S. Oke

**Affiliations:** 1 Centre for Invasion Biology, South Africa National Biodiversity Institute, Free State National Botanical Garden, Free State, Rayton, Dan Pienaar, Danhof, P.O Box 29036, Bloemfontein, 9310 South Africa; 2 South Africa National Biodiversity Institute, Free State National Botanical Garden, Free State, Rayton, Dan Pienaar, Danhof, P.O Box 29036, Bloemfontein, 9310 South Africa; 3 Faculty of Health and Environmental Science, Central University of Technology, Private Bag X20539, Bloemfontein, 9300 South Africa; 4 School of Life Sciences, University of KwaZulu-Natal, P/Bag X01, Scottsville, Pietermaritzburg, 3209 South Africa

**Keywords:** Avian frugivory, Grasslands biome, Seeds, Bush encroachment, Conservation

## Abstract

**Background:**

By transporting and scarifying the seeds during ingestion, avian frugivores reduce the competition with siblings, and may improve the germination which is critical for dispersal effectiveness and population recruitment. However, generally, there is limited knowledge on how deposited seeds interact/compete in the new microsite. We tested the hypothesis that the bird-dispersed seeds benefit from improved germination after their passage through the bird’s gut; and we investigated the potential impact of seed density on competition at the microsites by determining whether seed density and species diversity influence germination in the Free State Province, South Africa.

**Results:**

Overall, the results partly supported the hypothesis. Germination trials with defecated seeds of five plant species compared with the manually depulped seeds showed that only *Searsia lancea* had significantly higher seed germination success and improved germination speed after passage through the bird gut while *Ziziphus mucronata* only benefited rapid germination. There was a significant correlation between seed size and the germination of bird-ingested seeds except in *Olea subsp. africana* possibly due to possession of extremely hard protective seed cover*.* Seed competition experiments pointed to *Z. mucronata* and *O. subsp. africana* having significant germination performance that was positively correlated to seed density and seed size while *Ehretia rigida* did not germinate at all. Seed species diversity in the germination trays did not have a significant impact since the seeds of two former plant species consistently displayed significantly higher germination across the competition levels.

**Conclusions:**

We conclude that different plant species respond differently to seed ingestion by birds, and that further long-term tests for germination physiological responses of the seeds’ samples used in this study are required since poor germination observed in other tree/shrub species cannot be attributed to competition solely.

## Background

Seed dispersal is the movement of seeds away from their parent plants through a variety of dispersal vectors, either abiotic or biotic (Jones et al. 2017; Thomson et al. 2011; Wang and Smith 2002; Westcott and Graham 2000). Seeds in fleshy fruits (i.e., drupe) are dispersed or discarded, often after animals (e.g., birds) have consumed the soft edible portion of the fruit, called the mesocarp, and dispersed the ingested endocarp containing the seeds (Dardick and Callahan 2014). Seed dispersal is crucial in plant regeneration, since it promotes the maintenance of the genetic structure of many native plant species (Kleyheeg 2018; Egerer et al. 2018; Castilla et al. 2017; Howe 2016; Heleno et al. 2011). In addition, many native tree and shrub species require animal-mediated seed dispersal as a longevity strategy in response to environmental change (Howe 2016). Plants (i.e., angiosperms) interact with multiple mutualists in a network pattern to acquire a variety of ecological services (e.g., pollination and seed dispersal) (Jordano 2016; Mokotjomela et al. 2021; Thomas et al. 2007; Strauss and Irwin 2004). Avian frugivores have been consistently reported to be essential in shaping the seed dispersal pattern of native vegetation structures (Le Roux et al. 2020; Rehm et al. 2019; Trakhtenbrot et al. 2005; Jordano 2000). For example, Banos-villalba et al. (2017) reported that macaws dispersed 75% to 100% of seeds or fruits over ranges of up to 1200 m, and influenced the recruitment success of some forest plant species, thus shaping the vegetation structure and function of the Amazonian ecosystem, Beni Savannas.

The effectiveness of seed dispersal is a product of the quality and quantity components of the multiple vertebrate vector species that consume fruits and seeds of different plant species in each habitat (Mokotjomela et al. 2016; Schupp et al. 2010; Schupp 1993). The germination of ingested seeds represents the quality component, which is regarded as more influential in plant recruitment than the quantity component (Mokotjomela et al. 2016; Schupp et al. 2010; Schupp 1993). Avian frugivores occasionally modify the rate of germination and enhance the chances of seeds’ survival (Kleyheeg 2018; Mokotjomela et al. 2015, 2016; Thabethe et al. 2015; Fricke et al. 2013; Mokotjomela et al. 2021; Barnea et al. 1991). For example, Traveset et al. (2001) tested the germination characteristics of the seeds from different plants (*Rhubus ulmifolius, Osyris alba, Rubia peregrine, Asparagus acutifolius*, and *Phillyrea* spp) that passed through birds’ guts in the western Mediterranean shrubland, and found that different germination speeds/rates were promoted by the treatment in the birds’ gut. However, Alves-Costa and Eterovick (2007) investigated the seed/fruit trail of 53 species that were consumed and dispersed by coatis (*Nasua nasua*) in Mangabeiras Park, Southern Brazil, and found that the seeds’ passage through the coatis’ guts did not affect germination success, except for *Myrcia guajavaefolia*, which enjoyed a 50% increase in germination success. Also, Dlamini et al. (2018) compared the effect of frugivorous birds on the seed dispersal and germination of two alien invasive plants (*Schinus terebinthifolius* and *Listea glutinosa*) in South Africa, and found that frugivorous birds enhance the germination rate of *Schinus terebinthifolius* after seed treatment, although no positive effect was noted on the germination of *Listea glutinosa* after seed treatment.

The dispersal of large numbers of seeds over long distances from their maternal plants reduces intra- and inter-species competition (Mokotjomela et al. 2013; Schurr et al. 2009; Higgins et al. 2003), and provides essential genetic links between disconnected plant populations (Schupp et al. 2010; Nathan et al. 2008). Long-distance dispersal also increases the chances of recruitment in the absence of seed predators by maximising the seeds’ access to safe microsites (Howe 1986). In competitive conditions, the early dispersal of seed species leads to their occupation of safe microsites both above and below the ground, and consequently increases the species’ recruitment success (Korner et al. 2007). Plant species that germinate early may have an advantage in survival and growth, due to having more space and greater access to resources (Guido et al. 2017). The interspecific competition between seeds may differ from year to year, depending on the availability of resources and weather conditions, as well seed density in the microsite.

There are other factors that can cause variation in the seed germination success and the rate, such as seed size (Mandal et al. 2008), the seed’s endocarp (i.e., its hormones) (Negash 2010; Dalling et al. 2011; Bekele 2000), and the conditions where the seeds are deposited (Carlo and Tewksbury 2013; Hulme 1998). The size of the seed will interact with local abiotic conditions and other co-deposited seeds to influence germination. Studies have shown that plant species with large seeds have greater success in their germination rate, and a positive effect on the seedling’s survival than the small seeds (Kolodziejek 2017; Souza et al. 2014; Mandal et al. 2008; Kahmen and Poschlod 2008; Cordazzo 2002). Mandal et al. (2008) consistently found that variation in seed size influences the germination, after recording a 78% germination success for the large seeds of *Hyptis suaveolens*, while smaller seeds had a 41% of germination success. However, Moles and Westoby (2004) found no correlation between seed mass and the species’ germination of viable seeds. The seed endocarp provides physical protection against disease (Dardick and Callahan 2014), but conversely it is a barrier to the germination success (Negash 2010; Bekele 2000). For example, Negash (2010) and Cuneo et al. (2010) found that the hardened endocarp of *Olea europaea subsp. africana* reduced rapid germination by blocking moisture and oxygen from reaching the seed.

Seed germination success can be affected by among other factors, the competitive interactions between species as well as their density in the microsite (Orrock and Christopher 2010); and thus influence the ultimate individual plant species’ population fitness (Goets et al., 2018; Aschehoug et al. 2016; Johnson et al. 2008; Schupp 1993). It has been suggested that seeds of different species can sense each other prior to their emergence and shift their germination timing to ovoid competition (Ward 2016; Tielborger and Prasse 2009; Wang and Smith 2002). Depending on the species, seed germination may be either delayed or accelerated as a competitive strategy against the neighbouring seeds (Bergelson and Perry 1989; Lortie and Turkington 2002; Orrock and Christopher 2010; Turkington et al. 2005). Consistently, Orrock and Christopher (2010) observed an accelerated seed germination response in *Phytolacca americana* during situation of high seed density. It has been shown that the seeds endocarp of certain species modulates germination time using physiological mechanisms controlled by hormones that regulate seed dormancy and protect the embryo against pathogens by releasing the phenolic compounds (i.e., tannins) (see Raviv et al. 2017). In some cases, the released endocarp hormones can also suppress the germination of neighbouring seeds (Miransari and Smith 2014). Lortie and Turkington (2002) tested the effect of initial seed density on the structure of a desert annual plant community over three years. The results shown that emergence of seedlings was significantly influenced by initial seed density in all three years, with higher initial seed densities having lower rates of emergence (Lortie and Turkington 2002).

While it is relatively well-known how seeds are affected by passage through the gut and the associated variable retention times (Le Roux et al. 2020; Mokotjomela et al. 2015; Mokotjomela et al. 2021; Traveset et al. 2001), how post-dispersal seed predation affects recruitment (Hulme et al. 1998), and how scatter-hoarding also benefits species recruitment (Vander Wall and Beck 2012), limited attention has been given to how the seeds of different plant species that either remain at the deposition location or survive post-dispersal predation interact to complete the recruitment processes. Hulme (1998) argued that both variation in post-dispersal seed predation and differences in predation between plant species are important elements that facilitate the coexistence of different plant species, and at the same time seed predation directly influences competition for these microsites. We therefore postulated that, in areas where perching material for birds is rare—such as the grasslands-dominated habitats of the Free State Province, South Africa—seeds dispersed by birds accumulate in the areas they frequent most, thereby increasing the possibility of density-dependent competition—which, however, must be minimised by the long-distance dispersal of seeds. Studies have shown that the distribution of perching material, as well as selective fruit foraging by birds, influences the patterns of seed deposition (Aukema and Rio 2002; Howe 1986; Stiles and White 1986), with the perching points, and the most preferred fruiting plants receive large numbers of seeds (Carlo and Tewksbury 2013). In view of these, we tested the hypothesis that bird-dispersed seeds benefit from improved germination after their passage through the bird’s gut; and we investigated the potential impact of seed density on competition at microsites, determining whether species diversity influence competition with a focus on the fruit-bearing and avian-dispersed species of tree/shrubs in the Free State Province, South Africa.

## Methods and materials

### Study site

**Fig. 1:**
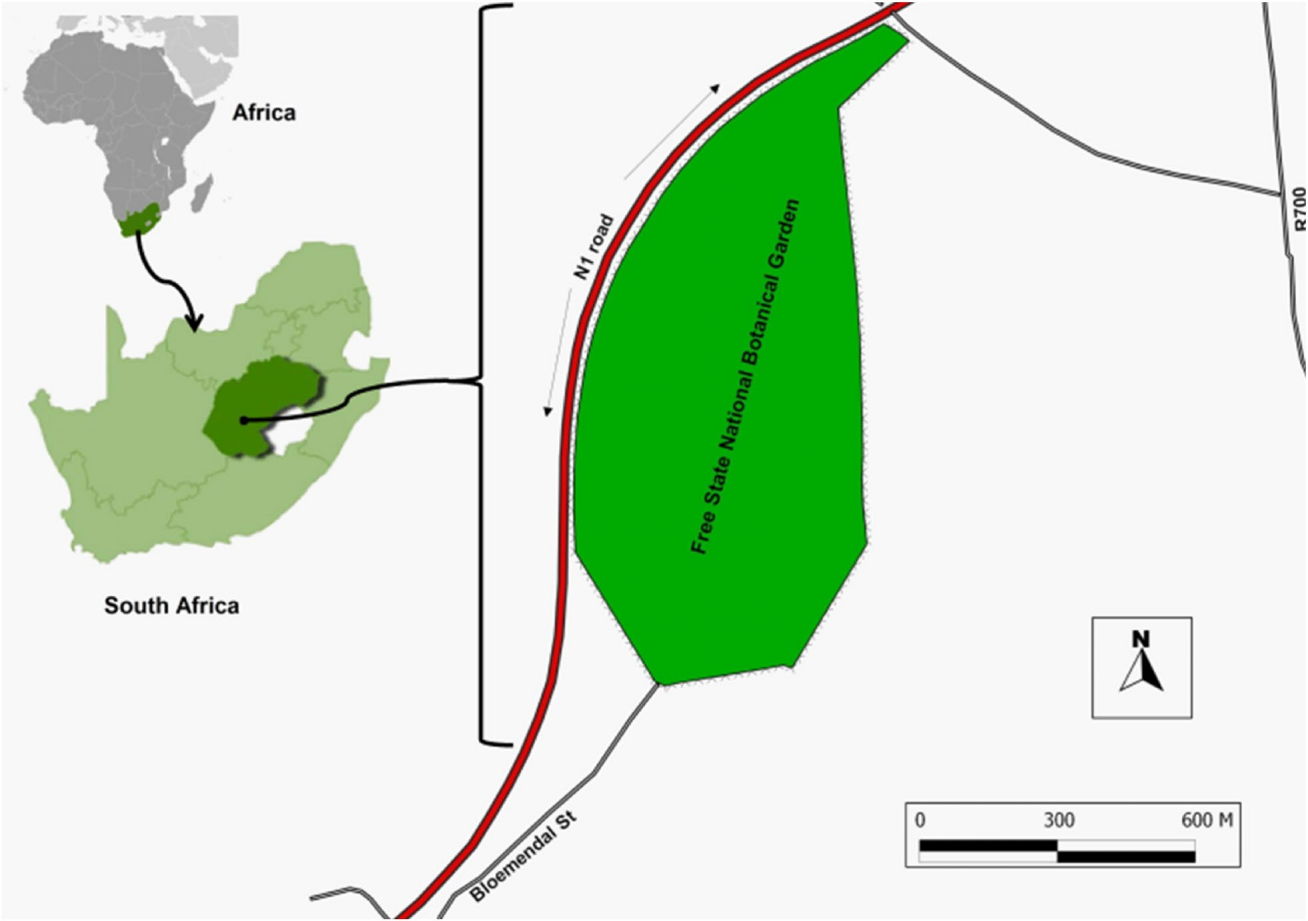
Location of the study area: Free State National Botanical Garden, Bloemfontein in South Africa

The study was conducted in the Free State National Botanical Garden (FSNBG) (*S 29° 12´ 55.6˝; E 26° 12´ 41,3˝),* Free State Province, South Africa (Fig. 1). The FSNBG covers an area of 66 hectares and is at an altitude between 1300 and 1400 m above sea level (Masilo 1999; Chaplin 1979). The vegetation structure is largely characterised by Bloemfontein Karroid Shrubland, Bloemfontein Dry Grassland, Winburg Grassy Shrubland, and Waterbody/Riparian vegetation (Mucina and Rutherford 2006). The FSNBG experiences a severe continental climate, with minimum winter temperatures below 0 ˚C, the night temperature often below freezing, and frost in July. Fairly high summer temperatures frequently range between 30 ˚C and 35 ˚C in January (Haddad et al. 2019; Neethling and Haddad 2013). The mean annual rainfall is relatively low (584 mm annually), with most of rainfall being recorded during the summer months and in early autumn.

### Seed collection and seed viability test

Seeds were collected on weekly bases during dry weather from various identified bird roosting areas (i.e., 17 locations) within FSNBG from March 2019 to February 2020. Only seeds that had been ingested and treated (i.e., that had passed through frugivorous birds’ gut and been trapped in the faecal samples) were collected. The seeds, which were free from insects and diseases, were stored in paper bags in a seed storeroom before the individual seed-counting and germination trials. We used the seeds’ structure (size, shape, coat, length, and width) to identify the different seeds (Ulian et al. 2019). For small seeds, we used the portable pocket 100× metal mini science microscope to identify each seed (Song et al. 2018). However, since moisture can disturb seed weight measurements, a digital calliper was also used to measure the length of each seed per species (i.e., 30 replicates) as a surrogate measure size.

Seed viability can be tested using the tetrazolium method, which was recommended by Van der Walt and Witkowski (2017) after applying it for a quick estimation of seed viability and vigour for *Adenium swazicum* in the Skukuza indigenous nursery, Kruger National Park. The tetrazolium test indirectly determines the respiratory activities in the cell found in the seed tissues (Franca-Neto and Krzyzanowski 2019). It is recommended that at least 100 seeds should be tested in replicates of 50 or less to increase the accuracy (Patil and Dadlani 2009; Delouche 1961). Due to the limited number of seeds, we did not experimentally test seed viability, but used the published results for similar species in South Africa.

The seeds of all the four species and the extra species were viable in various ways (Table 1). Two tree/shrub species, *Ziziphus mucronata* and *Searsia lancea*, displayed a viability of above 50%, while the other tree/shrub species (*Olea europaea subsp. africana*, *Grewia occidentalis*, and *Ehretia rigida*) had a seed viability that ranged from 8 to 45%.

**Table 1:** Seed viability (%) of different tree/shrub species that were included in the germination trials in this study

Species	Family	Seed viability %	Study area/country	Natural/ treated seeds	Source
*Ziziphus mucronata*	Rhamnaceae	71–100%	South Africa	Natural	Weiersbye and Witkowski, 2002
*Olea europaea subsp. africana*	Oleaceae	32–39%	South Africa	Natural	Mokotjomela et al. (2021)
**Grewia flava (c. G. occidentalis)	Malvaceae	29%	South Africa	Natural	Weiersbye and Witkowski, 2002
*Ehretia rigida*	Boraginaceae	8–45%	South Africa	Natural but sterilised	Wilman et al., 2014
*Searsia lancea*	Anacardiaceae	40–76%	South Africa	Natural	Weiersbye and Witkowski, 2002

Seed treatment and source of information are provided (** viability of another Grewia species was used)

*Ziziphus mucronata* is a small to medium shrub that fruit a shiny reddish to yellowish brown spherical drupe when ripe (March–august) with sub-globose of up to 36 mm in diameter (Van Wyk and Van Wyk 2013). The ripe fruits are edible and has a stony endocarp with seeds that usually solitary, elliptic, and compressed (Hassen et al. 2005).

*Searsia lancea* is a small to medium, hardy, frost- and drought-resistant evergreen tree that grow up to 7 m in height usually with single-stemmed (Van Wyk and Van Wyk 2013). The species’ fruits are slightly flattened, shiny spheroidal drupes of up to 5 mm in diameter that turns dull yellow to brown when ripe (Van Wyk and Van Wyk 2013), with the seed enclosed in an anacardium-type endocarp (Wannan and Quinn 1990).

*Olea europaea subsp. africana* is a small to medium evergreen tree which is drought- and frost-resistant and grow up to 12 m in height (Van Wyk and Van Wyk 2013). This species produces a small ovoid drupe fruit with spherical, thinly fleshy pulp which ripen purple-black (March—July), and seeds are indehiscent, stony, with hard endocarp (Cuneo et al. 2010).

*Grewia occidentalis* is a scrambling deciduous shrub that grow up to 3 m in height (Van Wyk and Van Wyk 2013). This species fruits (i.e., up to 1.5 cm wide) an edible distinctive four-lobed fruits common know as cross-berry or four-corner that turns shiny reddish-brown when ripe (January–May) with the seed enclosed in a pyrene stony endocarp (Van Wyk and Van Wyk 2013).

*Ehretia rigida* is a deciduous small, hardy and drought resistant shrub usually multi-stemmed that grow up to 9 m in height (Van Wyk and Van Wyk 2013). The species fruit a round edible fruit with orange to red, turning black when ripe and has a kidney shaped seed that is about 3 mm long (Retief and Van Wyk 2001).

### Seed germination success: ingested seed vs depulped seeds

One of the study’s hypotheses was that frugivorous birds modify the rate of seed germination for seeds ingested during foraging (Mokotjomela et al. 2015, 2016). To study the impact of birds’ ingestion of seeds on the germination success for plant species, we conducted seed germination trials, using the seed of four dominant native tree/shrub species: *Z. mucronata*, *O. subsp. africana*, *S. lancea*, and *E rigida*, whose seeds had passed through the gut of frugivorous birds. We also used manually depulped seeds of the same tree/shrub species as experimental control. All the ingested seeds were collected from the 17 birds’ roosting areas in the FSNBG.

We did not have appropriate infrastructure for keeping trapped birds for feeding trials in captivity, and thus research ethics could not endorse bird trapping. The surveillance digital camera traps (Uway VH200B), which are considered nonintrusive method of sample wildlife activities (Kays et al. 2011; Mokotjomela and Hoffmann 2013) were used to document bird species that defecated ingested seeds in different roosting sites during the peak fruiting time. Camera traps were set up following the bird foraging activity pattern: at sunrise (6h00), and monitoring at noon (12h00), and reset at 15h00 for afternoon. At sunset (18h00), image data was downloaded from cameras and occasionally, they were left in attempt to determine the nocturnal mammals that might provide secondary seed dispersal services to the deposited seeds.

**Fig. 2:**
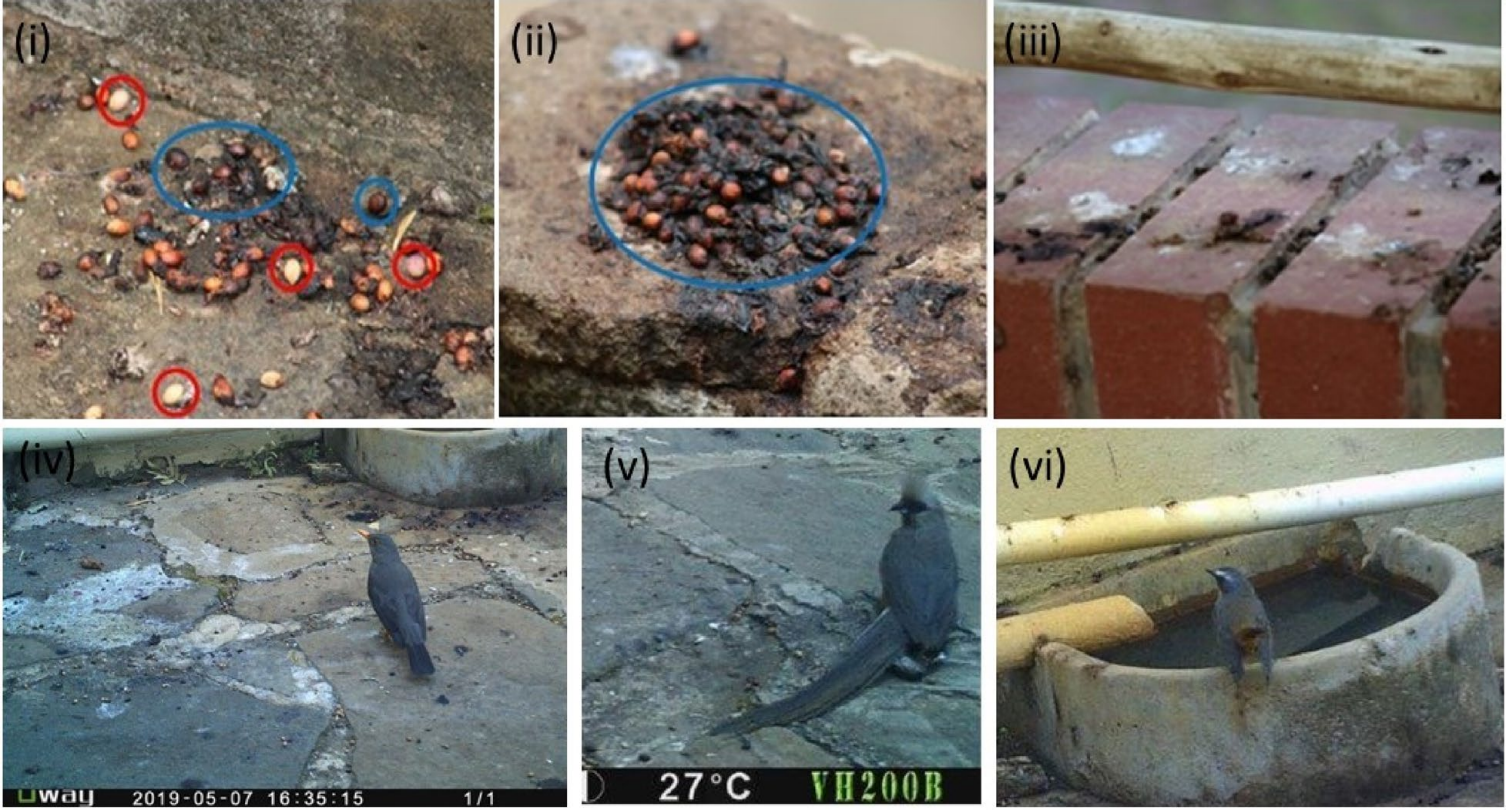
Birds roosting areas: faecal samples were collected during the study (i–iii). Red circle shows regurgitated seeds and blue circle shows defecated seeds. Common bird species that ingested and defecated intact seeds included (iv) olive thrush *Turdus olivaceus*, (v) speckled mousebird *Colius striatus* and (vi) cape robin-chat *Cossypha caffra* recorded in one roosting site in the study area

The three bird species namely: Olive thrush (*Turdus olivaceus*) (3.8 ± 0.8; N = 156; Fig. 2 iv), followed by the Cape robin-chat (*Cossypha caffra*) (1.4 ± 0.5; N = 156; Fig. 2 vi), and Speckled mousebird (*Colius striatus*) (1.0 ± 0.4; N = 156; Fig. 2 v) displayed highest daily visitation frequency.

For depulped seeds, ripe and fleshly fruits were harvested directly from four native tree /shrub species. All the fruits from the four plant species were soft and moist; the seeds’ chaff was rubbed off in water by hand, except for the fruits of *Z. mucronata*, for which a wire mesh screen was used: the fruit was rubbed off the seeds by moving them back and forth against the screen. A total of 100 seeds per plant species (50 ingested seeds and 50 depulped seeds) were used for the germination trials.

Before sowing the seeds, the trays were washed with household bleach to sterilise them against fungal attack and insect pests (Ulian et al. 2019; Mokotjomela et al. 2015). Forty trays were washed and placed in the sun to dry for a week before sowing (Nichols 2005). Each tray’s dimension was 16 cm × 14 cm × 4.5 cm; and each had drainage holes. The seeds were sown in the trays using 30 dm^3^ of potting soil, comprising milled pine bark, black loam, coarse river sand, and coconut palm fibre without any other components. Potting soil is a free-draining mixture that can hold moisture for the seed germination process to start (Nichols 2005). The potting soil was mixed with water to make it moist before it was placed in the trays. The seeds were sown at least 1–1.5 cm deep from the top of the tray to allow enough room during watering, and 2–4 cm apart, depending on the species (Ulian et al. 2019). For each plant species, 10 ingested seeds (for the treatment) and 10 manually depulped seeds (for experimental control) were sown in different trays, and each tray was replicated five times. Cotyledon emergence was recorded as successful germination on a daily basis, and the number of seedlings that emerged in each germination tray was recorded. Irrigation was done as necessary, either in the mornings before 10h00, or later, after 17h00. The seed germination trials were monitored over a period of at least 3 months, from November 2019 to the end of March 2020, to allow for possible delays in the seed germination of other species.

The study also hypothesised that variations in seed size (i.e., their weight and length) may influence their successful germination, with large seeds having a greater germination success than smaller seeds (see Souza et al. 2014; Mandal et al. 2008; Kahmen and Poschlod 2008; Cordazzo 2002). Therefore, the seed size of different tree/shrub species was measured. Because some seeds were too small for the scale, twenty seeds of each plant species were randomly selected and had their weight measured using a SWAN series digital scale in grams, which was replicated 30 times. Each measure of weight for 20 seeds was divided by the total number of seeds (i.e., 20) to get the weight of a single seed. Also, since moisture can confound seed weight, 30 seeds had their length as a surrogate measure of size measured using a digital calliper (i.e., the longitudinal dimension of each seed) for each plant species (Kleyheeg et al. 2018). Environmental conditions where seeds were collected, and the seed storage time had minimal impact on the results since almost all faecal samples was collected from dry surfaces and all seeds were sown within minimal dormancy time threshold (Table 2).

**Table 2:** Seeds attributes for different tree/shrubs that were included in the germination trails

Species	Family	Mean seed size ± standard error		Seed viability storage period (source)
		Seed length (mm)	Seed weight (g)	
*Ziziphus mucronata*	Rhamnaceae	8.72 ± 0.13 mm	0.19 ± 0.004 g	Seed viability improved with an age up to1 year in the dry storage (Weiersbye and Witkowski, 2002)
*Olea europaea subsp. africana*	Oleaceae	7.61 ± 0.14 mm	0.09 ± 0.003 g	Improves seed germination after being stored for 3 year (Fabbri et al., 2004)
*Grewia occidentalis*	Malvaceae	4.71 ± 0.13 mm	0.04 ± 0.002 g	**Grewia bicolor (c. G. occidentalis), seed can be stored up to 1 year before sowing (Heuzé et al., 2015)
*Ehretia rigida*	Boraginaceae	3.52 ± 0.10 mm	0.03 ± 0.007 g	** *Ehretia cymosa (c. E. rigida*) seed can be stored up to 12 months (Angaine et al., 2020)
*Searsia lancea*	Anacardiaceae	4.21 ± 0.05 mm	0.02 ± 0.001 g	Seed viability persist for up to 1 year (Weiersbye and Witkowski, 2002)

**Seed storage period of same genus was used

Germination speed is described as the of total number of the seedlings that emerged per time (Ranal and de Santana 2006; Maguire 1962). The seed germination speed was calculated as a ratio of the number of seedlings that germinated to the total number of days from sowing date to the termination of the germination trial (Mokotjomela et al. 2016).

### Interspecific seed competition across different densities between plant species

In this experiment, we investigated the competitive interactions during germination among seeds deposited by avian frugivores for five tree/shrub species that are likely to compete for dispersers’ vectors (i.e., birds): *Z. mucronata*, *O. subsp. africana*, *G. occidentalis* (seeds dispersed between March to July), and *S. lancea* and *E. rigida* (seeds dispersed between August and December). The hypothesis tested in this experiment was that the germination of bird-dispersed seed is free from competition near the maternal plant, and thus germinates much better than undispersed seeds. The bird-ingested seeds were sown in the trays (size: 16 cm × 16 cm × 4.5 cm), which were filled with potting soil and had drainage holes in the bottom. The seeds of each tree/shrub species were spread randomly in the trays and covered with a 1 cm layer of potting soil to mimic natural conditions (Nichols 2005). Species that were dispersed first (i.e., from March to July), were tested against each other first on the assumption that they would be the first to reach the microsite; and they were followed by two species that were dispersed between September and December. Since *Z. mucronata* displayed the highest level of germination in the first trials when testing the impact of passage through the gut, it was used as the reference species, and all the other species were added in decreasing order of seed size. Six replicates of each density set-up were sown: these entailed the low-density treatment of five seeds per species through to the high-density treatment of 20 seeds per species in each tray. Competing species (in groups of two, three, four, and five tree species) were sown according to four density classes at the same time: (1) six replicates of one container with five seeds per tree/shrub species; (2) six replicates of one container with ten seeds per tree/shrub species; (3) six replicates of one container with 15 seeds per tree/shrub species; and lastly, (4) six replicates of one container with 20 seeds per individual species. The seedlings’ emergence was recorded on a daily basis, and the number of seedlings in each tray was counted. Seed germination trials were monitored over a period of four months, from 22 November 2019 to 30 March 2020.

## Statistical analyses

### Germination trials: bird-ingested vs depulped seeds

To compare the germination rates between the bird-ingested seeds and the manually depulped seeds, the experimental design was balanced, with equal numbers of seeds in each tray. To test the hypothesis on the impact of the treatment of seeds during their passage through birds’ gut, the germination rate of the bird-ingested seeds was compared with that of the manually depulped seeds (i.e., the experimental control). The generalised linear model analysis of variance (GLM-ANOVA) with negative binomial was applied to determine the significant differences in the seed germinated for each tree/shrub species and treatments. The dependent variable consisted of the count of the number of seeds germinated, while the treatments (i.e., the bird-ingested and the depulped seeds) and the tree/shrub species were defined as predictor variables. The Statistical Package for Social Sciences (SPSS, version 20) software was used to perform the analyses.

Spearman's correlation was used to test for significant correlation between germination rate and seed size—i.e., length.

Since the daily germination counts lead zero-inflated count data, the weekly counts of germinated seeds were used to estimate the germination speed for the depulped and ingested seeds per tree/shrub plant species. The cumulative weekly germination data was plotted to generate the germination speed curves per plant species.

### Interspecific competition for germination of bird-ingested seeds among tree/shrub species

The seedling counts data were obtained from the balanced experimental design and we ran the comparisons of the seed germination for the different tree/shrub species across the different seed density levels and in the different combinations of tree/shrubs species per tray. Seedling counts data for competition level 1, was tested for normality using Shapiro–Wilk test (SW = 0.844, N = 49, *P* = 0.0001) since sample size was less than 50 (Zar 2010). To reduce the inequality of variance, the germination counts were log transformed prior statistical analyses. Due to the complexity of the experimental design, a General Linear Model—Factorial Analysis of Variance was applied to determine the significant differences in seed germination at different seed density levels and number of species (species diversity) using SPSS Version 20. The response variable consisted of the counts of the germinated seeds. The tree/shrub species and the seed density levels were defined as the predictor variables in order to allow an assessment of the interactions between the different combinations of plant species and the seed density per unit area. The Tukey HSD test was used to separate significantly different means at P ≤ 0.05. The same procedure was used to analyse data for other competition levels 2, 3 and 4.

Furthermore, for any tree/shrub species that showed significant difference during any competition level, an overall analysis was run to compare how the germination rate varied between different seed density using the nonparametric model Kruskal–Wallis test. Also, we determined if the germination rate has relationship with total numbers of seeds per tray using the Spearman rank order correlation.

### Germination of Z. mucronata and O. subsp. africana across the seed density levels

A nonparametric Kruskal–Wallis ANOVA was applied to compare the bird-ingested seed germination rate of *Z. mucronata* and *O. subsp. africana* across the different seed densities levels. The multi-comparison test distinguished different seed germination rates among the different seed density levels. Also, we tested for a correlation between germinated seeds of both plant species: *Z. mucronata* and *O. subsp. africana* and seed density (the total numbers of seeds per tray) to determine whether there was any relationship.

## Results

### Germination trials: bird-ingested vs manually depulped seeds

**Fig. 3:**
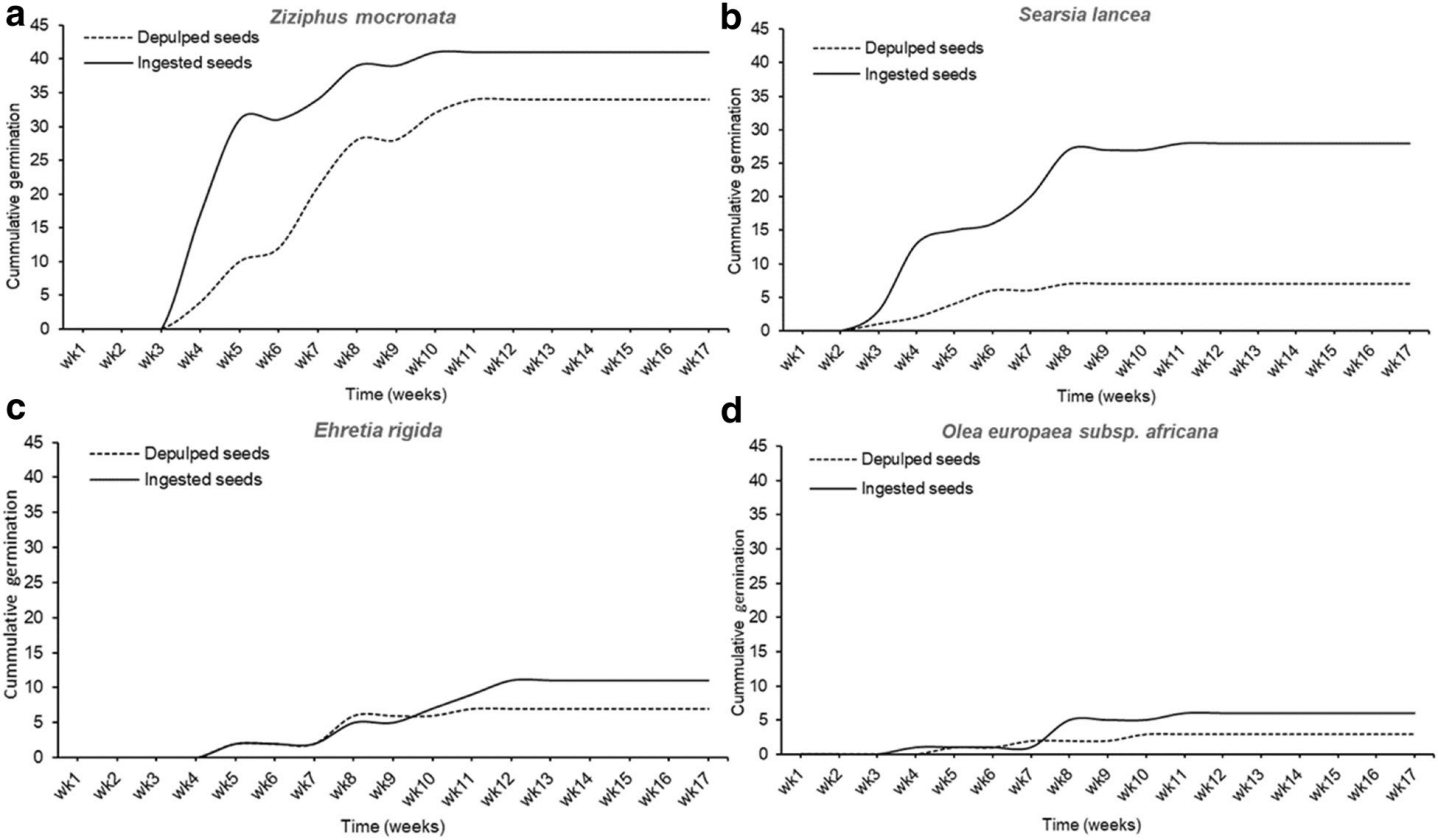
Cumulative weekly seed germination between bird-ingested seeds and manually depuplped seeds for four native tree/shrub plant species (**A** *Z. mucronata*, **B** *S. lancea*, **C** *E. rigida*, and **D** *O. supsp. africana*). The ingested seeds were extracted from bird faecal and depulped seeds were extracted direct from the tree/shrub ripe fleshly fruit

The comparisons of the germination rate between the bird-ingested and the manually depulped seeds were highly significant for the tree/shrub species (Wald χ^2^ = 142.9, df = 3, *P* < 0.001; Fig. 3). *Z. mucronata* had the highest germination rate for its seedlings (mean ± SE: 60.0 ± 8.0%; N = 20) (Wald χ^2^ = 55.0, df = 5, *P* < 0.001), but bird-ingestion did not improve seed germination (Wald χ^2^ = 1.4, df = 1, *P* = 0.226; Fig. 3). However, there were significant interactions between the seed germination of the different tree/shrub species and the seed treatments (Wald χ^2^ = 14.1, df = 3, p = 0.003; Fig. 3), with *S. lancea* having a much more significant germination rate for the bird-ingested seeds than for the manually depulped seeds (Wald χ^2^ = 12.7, df = 1, *P* = 0.001; Fig. 3B; Mean ± SE: 56.0 ± 5.0; N = 50 vs 14.0 ± 2.0; N = 50). The Turkey post-hoc test showed that there were no significant differences between the germination of the bird-ingested seeds and the depulped seeds of *O. subsp. africana* (Wald χ^2^ = 1.1, df = 1, *P* = 0.288; Fig. 3), and *E. rigida* (Wald χ^2^ = 0.3, df = 1, *P* = 0.619; Fig. 3).

*Searsia lancea* also had the seed germination speed improved significantly after passage through the bird gut (5.60 vs 1.44: Fig. 3B). Although *Z. mucronata* seed germinated was not improved by passage through the bird gut, the germination speed of ingested seeds was significantly improved (8.33 vs 4.69: Fig. 3A).

There was a significant correlation between seed size and the germination of bird-ingested seeds (Spearman Rank Order Correlation: r = 0.30; N = 640; *P* < 0.001), with the tree/shrub species that have large seeds having a greater germination than species with smaller seeds—except for *O. subsp. africana*.

### Interspecific competition for germination of bird-ingested seeds among tree/shrub species

**Fig. 4:**
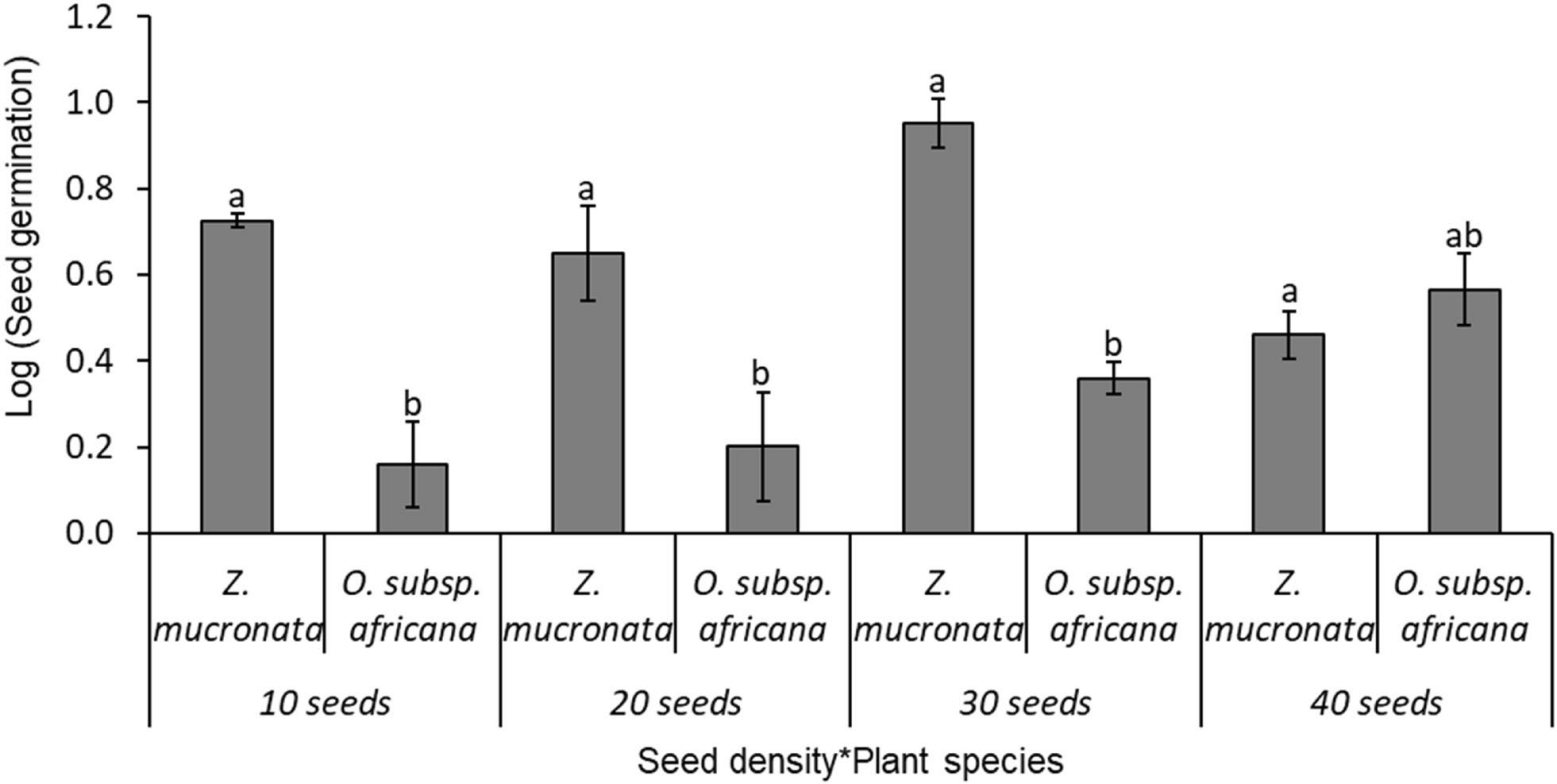
Mean seed germination for different plant species in competition level 1 between two tree/shrub species: *Z. mucronata* and *O. subsp. africana.* The error bars represent the standard error of sample mean, and different letters above standard error bar denote significant differences between seed density levels

**Fig. 5:**
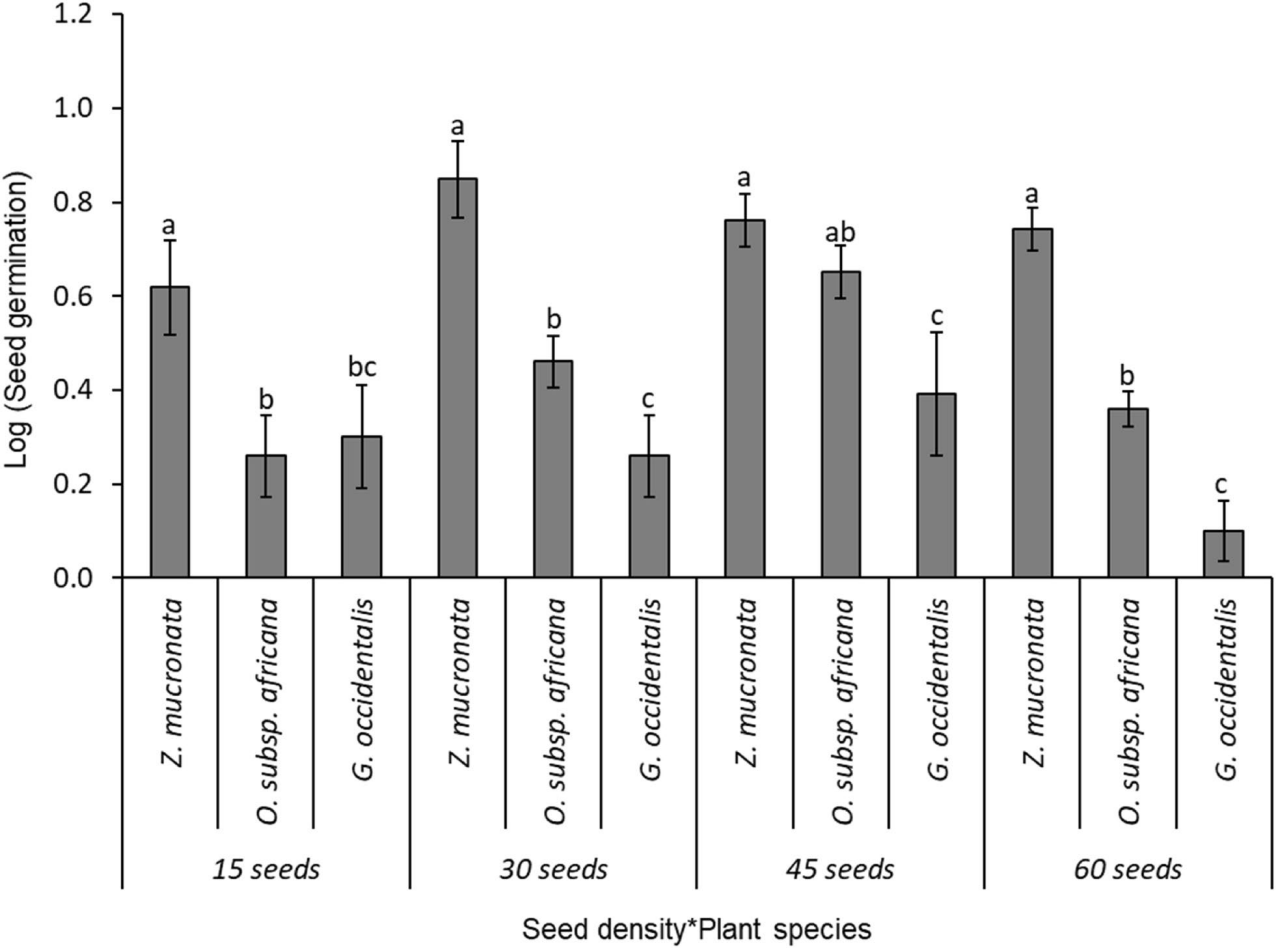
Mean seed germination for different plant species in competition level 2 between three tree/shrub species: *Z. mucronate*, *O. subsp. africana* and *G. accidentalis.* The error bars represent the standard error of sample mean, and different letters above standard error bar denote significant differences between seed density levels

**Fig. 6:**
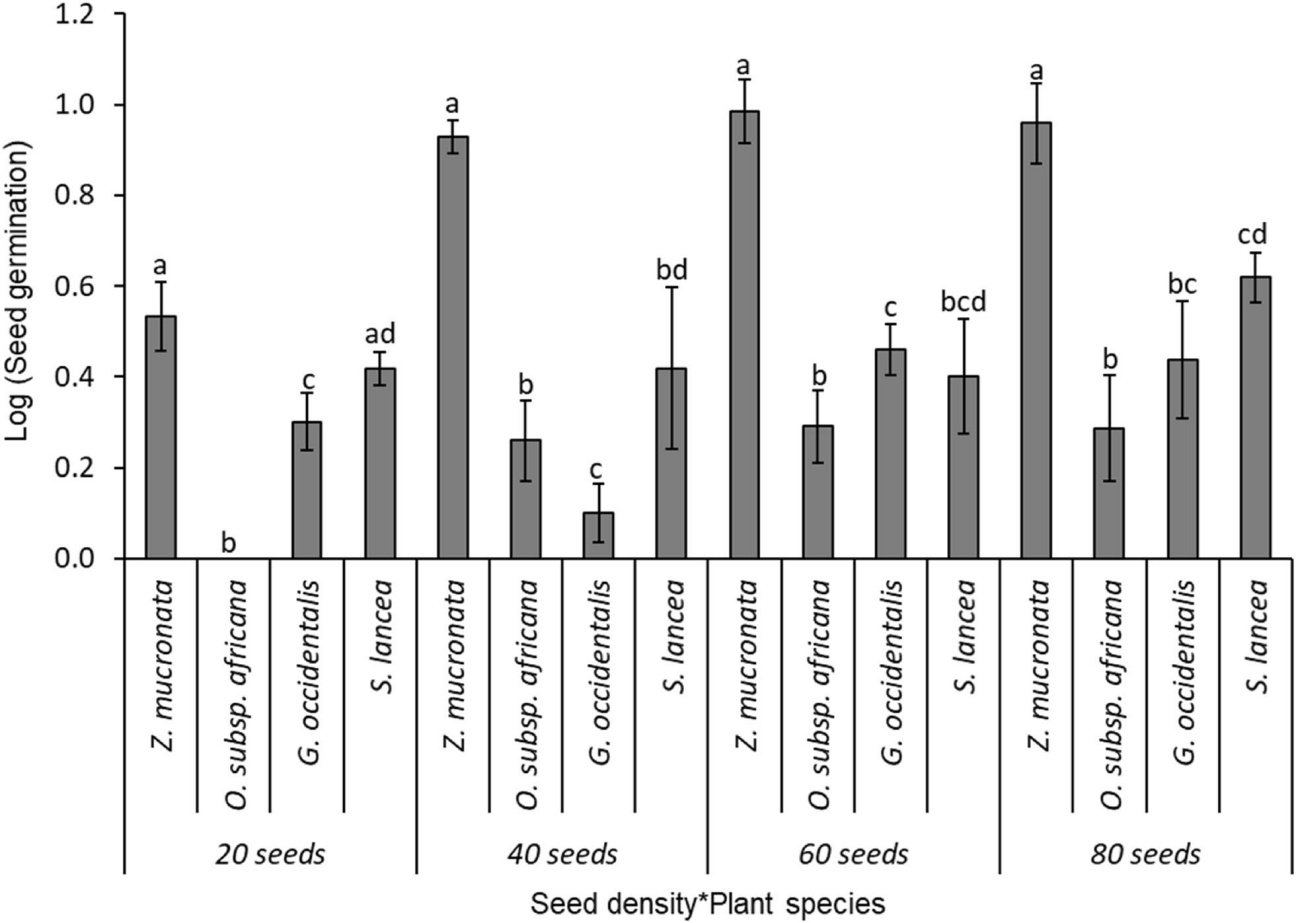
Mean seed germination for different plant species in competition level 3 between four tree/shrub species: *Z. mucronate*, *O. subsp. africana, G. accidentalis* and *S. lancea.* The error bars represent the standard error of sample mean, and different letters above standard error bar denote significant differences between seed density levels

(i) Level 1 competition test: two species (*Z. mucronata* and *O. subsp. africana)*
  The comparison of the bird-ingested seed germination between two competing tree/shrub species, *Z. mucronata* (mean ± SE: 48.9 ± 7.1%; N = 100), and *O. subsp. africana* (11.8 ± 4.8%), was highly significant (F _(1, 40)_ = 42.4; p < 0.001; Fig. 4). Similarly, different levels of seed density were significantly different in the seed germination of those two competing shrubs species (F _(3, 40)_ = 3.3, p = 0.030; Fig. 4). Significant interactions were observed between the seed density and the seed germination for the different tree/shrub species (F _(3, 40)_ = 8.0, p = 0.0003; Fig. 4), with *Z. mucronata* showing a significantly higher seed germination than *O. subsp. africana* across three seed density levels—at seed densities of 10 (86.7 ± 4.2%), 20 (43.3 ± 14.8%), and 30 (55.6 ± 7.4%)—but not at a seed density of 40 (10.0 ± 1.8%).
(ii) Level 2 competition test: three tree/shrub species (*Z. mucronata, G. occidentalis*, and *O. subsp. africana)*
  The comparisons of the bird-ingested seed germination rate among three competing tree/shrub species—*Z. mucronata* (mean ± SE: 49.2 ± 9.3%; N = 150)*, O. subsp. africana* (17.8 ± 3.9%), and *G. occidentalis* (12.9 ± 5.2%) was highly significant (F _(2, 60)_ = 36.2; p < 0.001; Fig. 5). The Tukey HSD test showed that, while the germination rate for *Z. mucronata* was significantly high, the latter two tree/shrub species had equivalent germination rates. Also, there were significant differences in the seed germination of the tree/shrub species across the different seed density levels (F _(3, 60)_ = 4.7; p = 0.054; Fig. 5). There were no significant interactions between the seed germination of the different tree/shrub species with the sown seed density levels (F _(6, 60)_ = 1.6; p = 0.153; Fig. 5), with *Z. mucronata* showing a higher germination than the other tree/shrub species across all seed densities: 15 (77.3 ± 16.9%); 30 (66.7 ± 12.8%); 45 (33.3 ± 4.9%) and 60 (23.3 ± 2.9%; Fig. 5).
(iii) Level 3 competition: four tree/shrub species (*Z. mucronata, G. occidentalis, S. lancea*, and *O. subsp. africana)*
  The comparisons of the bird-ingested seed germination rates among four competing tree/shrub species – *Z. mucronata* (mean ± SE: 59.9 ± 10.3%; N = 200)*, S. lancea* (23.3 ± 6.9%), *G. occidentalis* (12.5 ± 2.3%)*,* and *O. subsp. africana* (5.8 ± 2.1%); were highly significant (F _(3, 80)_ = 39.2; p < 0.001; Fig. 6), and there were significant differences in the seed germination across the different seed density levels (F _(3, 80)_ = 7.3; p = 0.0002). The Tukey HSD test showed that, while the germination rate for *Z. mucronata* was significantly high, *S. lancea* and *G. occidentalis* had equivalent germination rates, but they were significantly greater than those for *O. subsp. africana*. There were significant interactions between the tree/shrub species and the density levels (F _(9, 80)_ = 1.9; p = 0.051; Fig. 6), with *Z. mucronata* having a significantly higher seed germination rate in seed densities of 40 (76.7 ± 11.2%), 60 (64.4 ± 13.4%), and 80 (45.0 ± 9.1%) than the other shrubs, while *Z. mucronata* and *S. lancea* have greater seed germination percentages than the other two species at seed density of 20 (53.3 ± 11.2%; 33.3 ± 4.2%; Fig. 6).
(iv) Level 4 competition: five tree/shrub species (*Z. mucronata, G. occidentalis, S. lancea, E. rigida*, and *O. subsp. africana)*

**Fig. 7:**
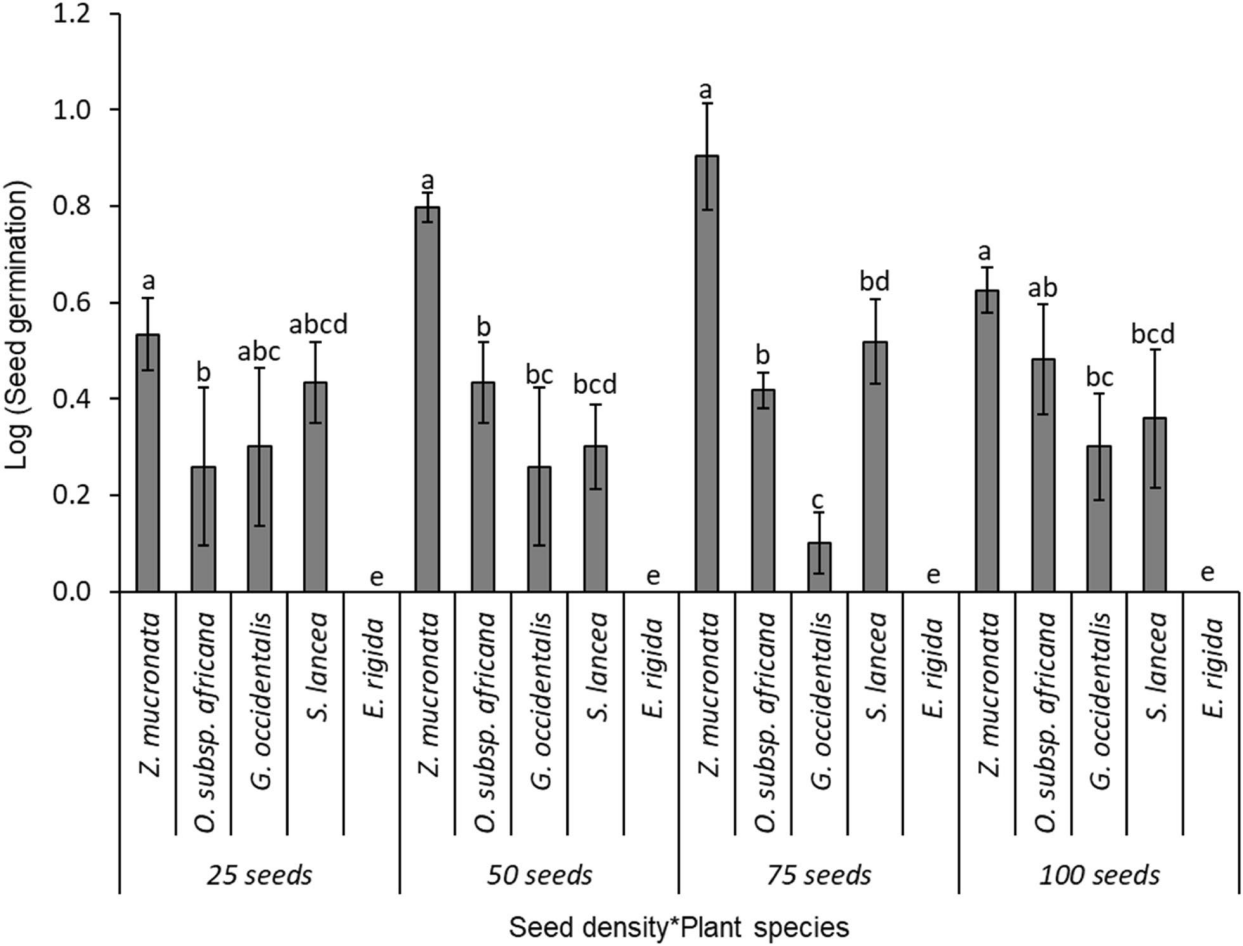
Mean seed germination for different plant species in competition level 4 between five tree/shrub species: *Z. mucronate*, *O. subsp. africana, G. accidentalis, S. lancea* and *E. rigida.* The error bars represent the standard error of sample mean, and different letters above standard error bar denote significant differences between seed density levels

The comparisons of the bird-ingested seed germination rates among five competing tree/shrub species – *Z. mucronata* (mean ± SE: 44.7 ± 8.1%; N = 250)*, O. subsp. africana* (21.1 ± 9.6%), *S. lancea* (19.4 ± 5.6%)*, G. occidentalis* (11.4 ± 3.7%)*,* and *E. rigida* (0.0 ± 0.0%); Fig. 7—were highly significant (F _(4, 100)_ = 35.8; p < 0.001; Fig. 7). The Tukey HSD test showed that, while *Z. mucronata* was significantly greater than other species, those for the next three tree/shrub species were not significantly different from one another but were significantly greater than for *E. rigida.* There were no significant differences in the seed germination at different seed density levels (F _(3, 100)_ = 0.7; p = 0.568; Fig. 7). There was no significant interaction between the seed density levels and the tree/shrub species (F _(12, 100)_ = 1.6; p = 0.114), while *Z. mucronata*, had the highest seed germination rate when the total seed density was 75 (55.6 ± 14.9%), and partly when the density was 50 (53.3 ± 4.2%; Fig. 7).

### Germination pattern of Z. mucronata and O. subsp. africana across the seed density levels

**Fig. 8:**
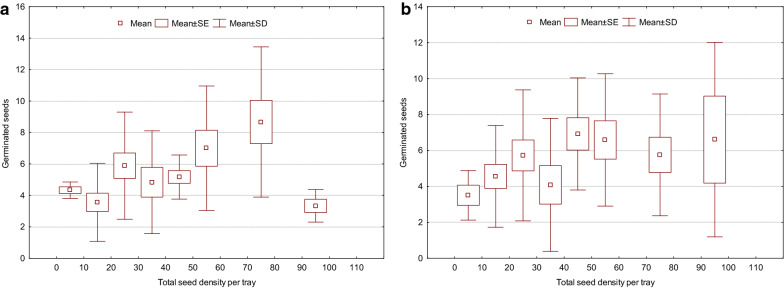
Overall variation in germination of bird-ingested seeds for *Z. mucronata* (**A**) and *O. subsp. africana* (**B**) across seed density levels (i.e., 10–100 seeds per tray) with different competing species

Overall, *Z. mucronata* was the strongest competitor against the other tree/shrub species, with significantly higher seed germination rates recorded across the different levels of seed density (H _(11, 96)_ = 30.5; *P* = 0.0013; Fig. 8A). The multiple comparison test showed that *Z. mucronata*’s seed germination was significantly (*P* = 0.0499) highest at the seed densities of 30 (mean rank: 67.8), 60 (61.8), 75 (63.8), and 80 (70.5), and these levels did not differ significantly (*P* > 0.05) among one other (Fig. 8A).

There was a significant positive correlation between the germination of the bird-ingested seeds of *Z. mucronata* and the seed density levels per tray (Spearman rank order correlation: r = 0.268; N = 96; *P* = 0.008).

Additionally, *O. subsp. africana* also displayed the significant increase in the germination rate as the seed density per tray increase (H _(11, 96)_ = 26.2; *P* = 0.0061; Fig. 8B). The multiple comparisons tests showed that these differences were recorded only at the seed density level of 45 with mean rank 81.8 against the seed density level of 20 with mean rank 24.7 (*P* = 0027). Similarly, the seed germination rate showed a significant positive correlation to the seed density (Spearman rank order correlation: r = 0.300; N = 96; *P* = 0.003).

## Discussion

### Germination trials: bird-ingested vs depulped seeds

We tested the hypothesis that the passage of seeds through birds’ gut improves germination and found—contrary to the hypothe—that bird-ingested seed germination improvement was not observed in four of the five tree/shrub species. Only *S. lancea* seeds had their germination improved by passage through birds’ gut, probably due to scarification of the seeds’ coat/endocarp in the avian digestive tract (Mokotjomela et al. 2015; Samuels and Levey 2005; Traveset et al. 2001), suggesting that this woody species benefits from a higher effectiveness of seed dispersal from birds than the others. In addition, the ingestion of *S. lancea* seeds by birds may release seed dormancy through the scarification associated with digestive processes (Mokotjomela et al. 2015; Traveset et al. 2001) and this also prevents the latent problems of either endogenous predation or disease that are reported in 60% of harvested seeds (Weiersbye and Witkowski 2002), thereby suggesting that *S. lancea* has a soft seed cover with limited resistance to microbial attack.

On the other hand, the unchanged germination rates of the bird-ingested seeds of *O. subsp. africana, E. rigida*, and *Z. mucronata* are likely the result of the high resistance of their seed cover to treatment in the gut, as reported by Mokotjomela et al. (2015) for the seeds of *Acacia cyclops*, in combination with the limited retention times when the seeds are ingested by large vectors (Tsoar et al. 2011; Schurr et al. 2009). The observed low germination of *O. subsp. africana* could be attributed to indehiscent, stony, and hard endocarp (Cuneo et al. 2010), which prevent germination by stopping moisture and oxygen from reaching the seed (Negash 2010; Bekele 2000). Also, *O. subsp. africana* seeds have a high seed dormancy that may persist for up to 35 months (Bekele 2000), which indicates uncertainty about the seed’s viability. The poor germination observed in *E. rigida* may be associated with an inherently low seed viability (~ 26%; Wilman et al. 2014), and a low tolerance to moisture and humidity (Louw 2012), whereas the study site has a humid microclimate due to the presence of a dam and wetland. This finding suggests that *E. rigida* is adapted to arid environments, as indicated by Wilman et al. (2014), and that it requires relatively low and intermittent moisture for successful seed germination (Wilman et al. 2014). We suggest that the unchanged but high seed germination in *Z. mucronata* is due to it having a large seed size that, reportedly (Mandal et al. 2008), also enhanced germination in the seeds of *Hyptis suaveolens*. This explanation can also hold for *Z. mucronata*, since we consistently found a significant positive correlation between seed size and germination rates. In addition, *Z. mucronata* has a high seed viability of up to 100% (Weiersbye and Witkowski 2002) and a high physiological plasticity in a broad variety of environmental conditions (Refka et al. 2013; Zietsman and Botha 1987). An unchanged germination of bird-ingested seeds has been previously reported in South Africa (Mokotjomela et al. 2015; Chama et al. 2013; Mokotjomela et al. 2021; Jordano et al. 2016), which highlights, in this context, the role of birds in transporting seeds to new microsites. However, we argue that these tree/shrub species could have evolved resistant seed covers in order to endure environmental uncertainties such as rodents’ seed predation, diseases, and droughts (Pearson et al. 2014; Dardick and Callahan 2014). Thus, further investigation that is beyond the scope of this study might yield better insight into the roles of the reported characteristics in the life of seeds in the local context.

Only *S. lancea* and *Z. mucronata* have their seed germination speed significantly accelerated, suggesting that those tree/shrub species benefited from scarification of the seeds’ coat in the avian digestive tract (Mokotjomela et al. 2016; Traveset et al. 2001), of which likely released seed dormancy (Vellend 2010). Seed abrasions and scarification by birds increase seed coat permeability and thus, quick imbibition (Mokotjomela et al. 2016; Figueroa and Castro 2002). Also, the medium-size bird Olive thrush (*Turdus olivaceus)* was most dominant bird in the study site, and Godínez-Alvarez et al. (2020) showed that the medium birds have longer gut tract retention time for seeds of which is critical for seed treatment. Consistently, the blackbirds (*Turdus merula)* are known to improve seed germination for woody plant species elsewhere (Barnea et al. 1991). Our results were similar with the findings of Mokotjomela et al. (2016), who reported that the bird’s gut treatment accelerated seed germination speed of the two cacti species in South Africa. Accelerated seed germination provides a competitive advantage of low seedlings’ competition for resources and thus promote plant species recruitment.

### Interspecific competition for seed germination: seed density and species diversity

Germination is a key process for maintaining plants’ genetic material in order to sustain species’ population after their seeds are dispersed to new microsites (Carlo and Tewksbury 2013; Hulme 1998). When seeds of different plant species interact, their ability to affect and respond to competition determines the contribution of seed germination to individual plant species’ population (Bergelson and Perry 1989; Lortie and Turkington 2002; Orrock and Christopher 2010; Tielborger and Prasse 2009; Schupp 1993). Part of this study investigated how bird-dispersed seeds handle competition mediated through seed density and species diversity in the microsite.

Our finding that *Z. mucronata*’s seed germination performance increased with seed density and species diversity may be due to its high behavioural plasticity (Refka et al. 2013; Zietsman and Botha 1987), an attribute that has possibly allowed *Z. mucronata* to endure emerging physiological conditions when competing for survival with other species. Similar results were reported by Orrock and Christopher (2010), who observed an accelerated germination response of *Phytolacca americana* when seed density was increased. Indeed, dispersed seeds may have to overcome a variety of environmental pressures against germination to attain their subsequent plant recruitment (Orrock and Christopher 2010; Wang and Smith 2002). If the experimental germination trays represented a microsite that was conducive to dispersed seeds, as proposed by Howe (1986), then – in line with the enemy release hypothesis (i.e., for invader species), which postulates that new environments have either few or less competitive neighbours (Le Roux et al. 2020), we argue that this could have enhanced *Z. mucronata*’s seed germination. Our study also highlights that the number of seeds (i.e., the amount of dispersal effectiveness, sensu*,* Schupp et al. 2010) in the microsite is important for the effective initiation of the recruitment process for each plant (Hulme 1998; Howe 1986) because, at a seed density of between 60 and 100, there was no significant increase in germination. Seeds’ endocarp has some hormones that play a major role in maintaining seed dormancy and in protecting the embryo against pathogens by producing phenolic compounds (i.e., tannins) (Raviv et al. 2017). If the seeds of different species can sense each other prior to their emergence (Ward 2016; Tielborger and Prasse 2009), it is possible that the endocarp of the germinated seeds of *Z. muconata* released chemicals and/or hormones that eventually suppressed the germination of neighbouring seeds, while it enjoyed all the germination resources available at the microsite. The impact of the suggested chemical effect could be amplified by the large seed size of *Z. mucronata.*

The fact that *O. subsp. africana* displayed an increasing seed germination in the level two competition (i.e., a total seed density of 15–45; the competing species were *Z. mucronata* and *G. occidentalis*), suggests that there could have been a physiological enhancement from the seeds of other species, since Negash (2010) and Cuneo et al. (2010) found that the hard seed endocarp of *O. subsp. africana* thwarts the germination process. It has been consistently shown that dispersed seeds adjust their dormancy status in relation to the new conditions in the microsite, while also targeting the optimal conditions for germination (Footitt et al. 2018), and that they can sense the presence of seeds of other species (Tielborger and Prasse 2009). Because the passage of seeds through birds’ gut did not also improve the germination of *O. subsp. africana,* we argue that the disintegration of the endorcarps of the substantially germinated seeds of *Z. mucronata* might have resulted in new physiological effects that favoured seed germination in *O. subsp. africana.*

The poor seed germination of *G. occidantalis* and the failed germination of *E. rigida* (i.e., in competition Level 3: species: *Z. mucronata, O. subsp. africana, G. occidantalis*, and *S. lancea*; and Level 4: species: *Z. mucronata, O. subsp. africana, G. occidantalis, S. lancea*, and *E. rigida*) could be partly attributed to the adaptive mechanisms related to a need for the persistence of dispersed seeds through protection against microbes and physiological barriers to their germination (Dalling et al. 2011). Orthodox seeds tend to dry up to preserve their viability (Hilhost and Toorop 1997), and this requires some treatment to break their dormancy (Qasem 2019; Rowarth et al. 2007). Bewley and Nonogaki (2017) distinguished between the sequence of the different physiological stages of the germination process, and they contended that, during the imbibition phase of germination in dry seeds (i.e., orthodox), germination may be prolonged if all the essential protein are not available to reinitiate the metabolism in the dormant seeds. Moreover, it has been shown that dispersed seeds undergo a transition of dormancy that is mediated by the maternal plant to an independent seed-mediated dormancy, which is also aligned with the new conditions at the location where the seed is deposited (Finch-Savage and Footitt 2017; Footitt et al. 2013). Thus, it is possible that the seed germination of the species in our study that were dispersed between March and July was retarded by the dormancy transition mentioned above. The emergence of the seed in some species may not be necessarily because of competition, but also because of the factors were not controllable such as fruit maturing time and their consumption by birds although all seeds were sown at age before dormancy was changed.

Recalcitrant seeds must be sown immediately after harvesting or being dispersed (Barbedo et al. 2013), unlike orthodox seeds (*Z. mucronata, G. occidentalis, O. subsp. africana*, *and E. rigida*). We suggest that the poor seed germination performance in *S. lancea*, as a recalcitrant seeder, could be partly due to the loss of its viability through dehydration during storage, which corroborates a report that *S. lancea* seeds are sensitive to moisture and thermal stress (Nichols 2005). Furthermore, this observation may be explained by the small seed size of *S. lancea*, which reportedly promotes persistence in the soil better than is the case with large seeds (Mole et al. 2000; Funes et al. 1999).

#### Concluding remarks

Our germination trials with the bird-ingested seeds extracted from faecal samples showed that the foraging birds provide seed dispersal service in two different ways relating to quality and quantity of dispersal effectiveness (Kleyheeg et al. 2018; Mokotjomela et al. 2016; Schupp et al. 2010). These are the partly improved seed germination success and its rate after passage through the bird gut; and solely, the transport of the seeds to new microsites. Although there was limited improvement seed germination success restricted to *S. lancea*, we noted that seed germination speed increased in *S. lancea* and *Z. mucronata* of which may provide competitive advantage for seedlings.

Foraging birds disperse the ingested seeds away from their maternal plant to the new microsite where there is less intra- and inter-species competition for resources that might be limited where siblings’ and congeners’ populations are large (Howe 1986). However, the results of this study suggest that beyond the seed dispersal and deposition, interspecific seed competition for germination resources still occur. The reported competition has implications for dispersal effectiveness since not all dispersed seeds germinated. We speculated that a prolific performance of *Z. mucronata* and *O. subsp. africana* is partly due to the reported behavioural plasticity in combination with seeds’ physiological defence to the competitors or change in the critical environmental conditions such as moisture in germination trays*.* As we cannot conclude that seed emergence of other species was due to inability to compete, further, studies on the physiological interactions of seeds underground are needed for elucidating on the germination behaviour of individual focal tree/shrub species. Finally, the high seed germination of the large seeded *Z. mucronata* in this study, explains its predominance in the reported bush encroachment in the study site (Vukeya et al. 2020; Department of Environmental Affairs, 2019; Zietsman et al. 1989), thus we recommend adaptive conservation management plan should include monitoring and prioritised population control for this species to maintain ecological balance with other species.

## Data Availability

Not applicable.
